# Determination of the Presence of Three Antimicrobials in Surface Water Collected from Urban and Rural Areas

**DOI:** 10.3390/antibiotics2010046

**Published:** 2013-02-07

**Authors:** Alejandra Iglesias, Carolina Nebot, Jose M. Miranda, Beatriz I. Vázquez, Carlos M. Franco Abuín, Alberto Cepeda

**Affiliations:** Department of Analytical Chemistry, Nutrition and Bromatology, Faculty of Veterinary Medicine, University of Santiago de Compostela, 27002, Lugo, Spain; E-Mails: alejandra.iglesias@usc.es (A.I.); josemanuel.miranda@usc.es (J.M.M.); beatriz.vazquez@usc.es (B.I.V.); carlos.franco@usc.es (C.M.F.A.); alberto.cepeda@usc.es (A.C.)

**Keywords:** drug, residue, antimicrobial, LC-MS/MS, surface water, rural, urban

## Abstract

Due to the continuous release of antimicrobials into the environment, the aim of this study was to compare the frequency of detection of sulfamethazine, sulfamethoxypyridazine and trimethoprim in surface water collected from urban and rural areas in Northwestern Spain. A monitoring study was conducted with 314 river water samples analyzed by high-performance liquid chromatography coupled to tandem mass spectrometry. The results indicated that 37% of the samples contained residues of at least one of the investigated antimicrobials, and every sampling site yielded positive samples. At sites located near the discharge points of wastewater treatment plants and near the collection point of a drinking-water treatment plant, more than 6% of the samples were positive for the presence of antimicrobial residues.

## 1. Introduction

In the last decade, interest in the occurrence and fate of pharmaceutical compounds in the aquatic environment has grown significantly due to the exponential increase in the global production and consumption of these compounds [1]. The main concern is that regardless of what drug is administered, once the drug has been metabolized, part of the initial dose is excreted in feces and urine in its original form and/or as metabolites. Through the domestic wastewater system, pharmaceutical compounds used in human medicine are conducted to wastewater treatment plants (WWTPs), where they should be removed. However, studies have demonstrated the presence of these compounds in the final effluents discharged by WWTPs and, consequently, their introduction into the aquatic environment [2]. Veterinary drugs used in animal production may be excreted directly into the environment or accumulated in manure pits. The application of manure to agricultural land as fertilizer may be another route through which active compounds are introduced into the environment [3,4,5]. 

The environmental persistence, rate of spread and bioaccumulation ability of biologically active substances differ depending on their chemical properties and on the environmental conditions. The continuous input of these compounds into the environment may lead to ecotoxicological effects [4,6,7]. In particular, antimicrobials are one of the most important groups of pharmaceuticals, employed in both human and veterinary medicine. These compounds have been used in large quantities for decades, and the emergence of antimicrobial resistance has prompted researchers to investigate their presence in the environment [8,9]. Such active compounds are frequently detected in environmental water samples. Data collected from different countries (USA, UK, Belgium, Croatia, India, Japan, Spain, Portugal) have revealed concentrations of pharmaceuticals in various aquatic environments ranging from the ng·L^−1^ to the mg·L^−1^ [10,11,12,13,14,15,16,17,18]. These differences in drug concentrations may depend on the matrix, sampling site, date and weather conditions [19]. Importantly, resistant bacteria might also reach the food chain and affect human health, especially via drinking water [20].

Sulfonamides are the most commonly used antimicrobial group in human and veterinary medicine. The main advantage of this family of compounds, aside from their relatively low cost, is that they provide a broad spectrum of action, affecting a variety of micro-organisms (Gram-positive and Gram-negative bacteria, *Chlamydia* and some protozoa), interrupting folic acid synthesis and preventing micro-organismal multiplication. These antimicrobials are commonly combined with trimethoprim, which inhibits protein synthesis, due to their synergistic effects [21]. 

This study focuses on the occurrence of two sulfonamides (sulfamethazine and sulfamethoxypyridazine) and trimethoprim in surface water. Previous studies have investigated the presence of these drugs in the aquatic environment [5,11,14,22,23,24,25]; however, the aim of the present study was to compare their frequency of detection in urban areas and rural areas (with and without farming activities) in the largest Galician river, the Miño River, in Northwestern Spain. A total of 314 river water samples were analyzed by high-performance liquid chromatography coupled to tandem mass spectrometry (HPLC-MS/MS). 

## 2. Results and Discussion

### 2.1. Concentrations of Pharmaceutical Compounds in Surface Water Samples

**Figure 1:**
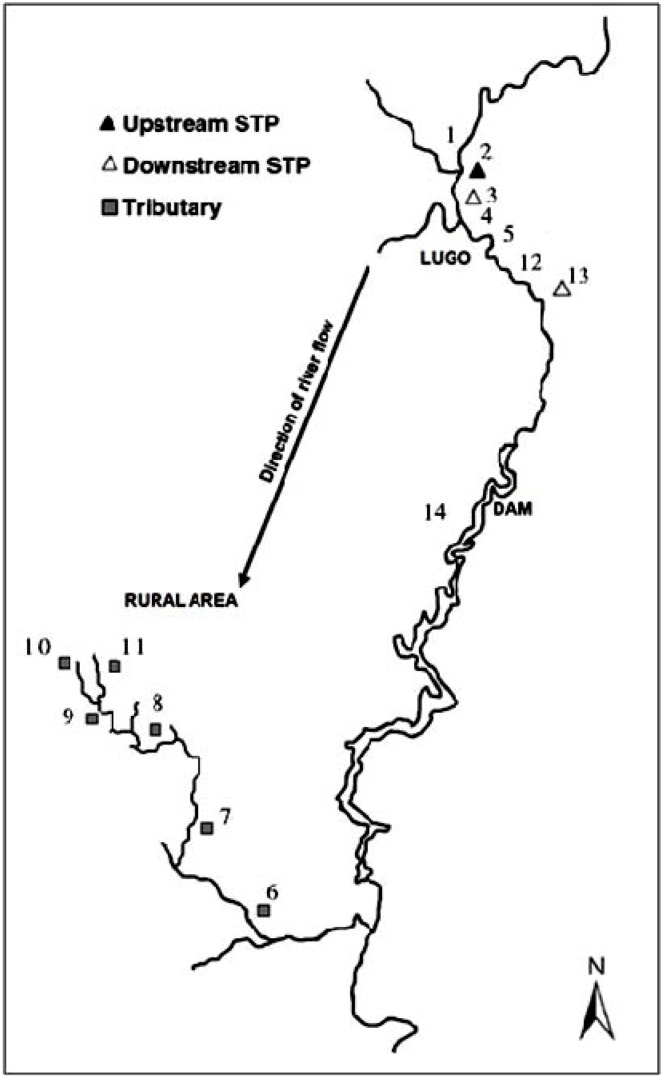
Graphic representation of the study area.

The limits of detection (LOD) and limits of quantification (LOQ) of the method and the maximum, minimum and mean concentrations and detection frequencies of the investigated drugs in the 314 surface water samples are summarized in Table 1. Site numbers and characteristics are specified in Table 2 and Figure 1.

**Table 1:** Chemical properties, limits of detection (LOD), limits of quantification (LOQ) of the method, maximum, minimum and mean concentrations and detection frequencies of the investigated drugs in surface water samples.

Analyte	Sulfamethazine	Sulfamethoxypyridazine	Trimethoprim
**MW**	278.3	280.3	290.3
**Formula**	C_12_H_14_N_4_O_2_S	C_11_H_12_N_4_O_3_S	C_14_H_18_N_4_O_3_
**Chemical structure**	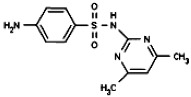	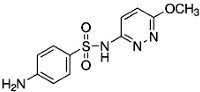	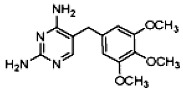
**LOD (ng·L^−1^)**	0.2	0.1	0.2
**LOQ (ng·L^−1^)**	0.5	0.5	0.5
**Maximum concentration (ng·L^−1^)**	63.6	11.2	85.4
**Minimum concentration (ng·L^−1^)**	0.5	0.5	1.1
**Mean concentration (ng·L^−1^)**	5.5	1.6	13.8
**Number of detections**	62	37	43

MW: Molecular Weight.

**Table 2:** Site number (Figure 1) and type, number of samples analyzed and number of positive sample of each sampling site.

Site number	Area type	Total number of samples analyzed	Number of positive samples
1	Rural without much farming activity	24	8
2	Rural without much farming activity	24	9
3	Urban	24	17
4	Rural without much farming activity	24	5
5	Rural without much farming activity	24	7
6	Rural with farming activity	24	4
7	Rural with farming activity	24	6
8	Rural with farming activity	24	9
9	Rural with farming activity	24	6
10	Rural with farming activity	24	8
11	Rural with farming activity	24	6
12	Urban	24	6
13	Urban	24	22
14	Urban	2	2

The results indicated that 37% of the samples collected were positive for the presence of at least one of the three drugs investigated. Among these positive samples, 34% were collected from rural areas dedicated to farming activities, and 66% were collected from urban areas or rural areas with no farming activities. Figure 1 indicates the location of the urban sampling sites (points 3, 12, 13 and 14), rural with not much farming activity sites (points 1, 2, 4 and 5) and rural farming sampling sites (points 6, 7, 8, 9, 10 and 11) selected for this study, which had the mean percentages of positive samples of 10, 6 and 5%, respectively. Although more positive samples were expected in agricultural areas, because large quantities of veterinary pharmaceuticals are used in food production [26], these results indicated that human pharmaceuticals are concentrated in areas near WWTPs. The opposite effect was observed for veterinary pharmaceuticals, which appeared to be dispersed directly into the environment in the study area. The selected drugs, sulfamethazine, sulfamethoxypyridazine and trimethoprim, are antimicrobials commonly used in human and veterinary medicine due to their price and broad spectrum of activity. Besides that these three antimicrobials are employed to the treatment of different bacterial infections, between 60%–80% of the initial dose is excreted via urine in both animal and human, explaining their high detections after the discharge of the WWTPs.

Positive samples were detected at all sampling sites (Table 2); indeed, more than 3% of the samples collected at each site were positive for at least one pharmaceutical. Overall, sampling sites located downstream from WWTP discharge points, sites number 3 and 13, yielded the largest number of positive samples, 17 and 22, respectively; more than 14% of the samples were positive. The literature provides evidence that WWTPs are point sources of pharmaceuticals in the aquatic environment due to the inefficient removal of these compounds during wastewater treatment processes [27,28,29,30,31]. Samples obtained from a tributary of the Miño River (point 6) had the lowest percentage of positive samples (3%). No clear explanation of this result was found; samples obtained from brooks and streams that discharge into this tributary yielded more than 5% of positive samples. 

Notably, samples obtained near the collection point of a drinking-water treatment plant (point 5) were positive for the presence of at least one of the analyzed antimicrobials. Seven positive samples were detected from this point (6% of the samples collected) (Table 2), with a maximum concentration of 56.3 ng·L^−1^ for trimethoprim. If the drinking-water treatment plant does not fully remove these antimicrobial residues, antimicrobials may be present in the water supplied to the human population, possibly leading to food-safety problems due to the long term exposure to low concentrations. Therefore, the presence of sulfamethazine, sulfamethoxypyridazine and trimethoprim should be investigated in the drinking water supply in the study area. 

Sulfamethazine, sulfamethoxypyridazine and trimethoprim were detected in 54%, 32% and 37% of the positive samples, respectively. Sulfamethazine, the most frequently detected drug—62 samples—had a maximum concentration of 63.6 ng·L^−1^ (Table 1), but trimethoprim had the highest maximum concentration of any drug measured in this study—85.4 ng·L^−1^—detected in 43 samples (Table 1). Sulfamethoxypyridazine was detected in 37 samples with a maximum concentration of 11.2 ng·L^−1^ (Table 1). The detection frequency and maximum concentration of trimethoprim obtained here were similar to those reported by Conley [19] in the Tennessee River; however, the mean concentration obtained in that study was half that measured here. Other studies that have investigated the presence of trimethoprim in the aquatic environment have obtained similar results of concentration and detection frequencies [13,14,32].

García-Galán [25] reported lower concentrations of the two sulfonamides in the Ebro River compared to those observed here, possibly due to a dilution effect resulting from higher river flow in the Catalonian samples [33,34], to the effect of sunlight [35], to lower consumption or to greater removal efficiency. However, Díaz-Cruz [22] detected sulfamethazine and sulfamethoxypyridazine concentrations of up to 12 µg·L^−1^ in surface water from the Llobregat River, collected at a sampling point downstream from agricultural areas.

### 2.2. Statistical Results

A standardized bias or standardized kurtosis outside the range of −2 to +2 for the compared factor levels indicated significant non-normality in the data. Thus, one-way ANOVA could not be employed. Therefore, the Kruskal-Wallis test was used to compare the median values instead of the means. There were statistically significant differences with a 95.0% confidence level (*p*-values less than 0.05) between the median concentrations of sulfamethazine and sulfamethoxypyridazine by sampling date, between the March and May sampling dates for sulfamethazine and between the November and December sampling dates for sulfamethoxypyridazine. Sulfamethoxypyridazine concentrations also differed significantly with solar irradiation, temperature and humidity during November and December. This observation was already described by Vieno [29], who concluded that winter conditions increased the detected levels of pharmaceuticals due to the lower temperatures, which reduce biodegradation. Although sulfamethazine concentrations also differed significantly with solar irradiation, temperature and humidity, only solar irradiation and sampling date corresponded to the same sampling period. Trimethoprim concentration did not differ significantly with weather conditions or sampling date, in contrast to the results of Hua [30], who reported higher concentrations of this compound in early spring. On the other hand, the trimethoprim and sulfamethoxypyridazine concentrations showed significant differences based on the physical and chemical parameters analyzed (nitrites, ammonium, conductivity, turbidity and pH). Sulfamethazine concentrations did not show significant differences based on ammonium and nitrite levels.

The concentrations of trimethoprim and sulfamethoxypyridazine differed significantly among sampling sites; the points located downstream from WWTPs discharges had the greatest number of positive samples. 

## 3. Experimental

### 3.1. Study Area

The study area was located in the Miño River and one of its tributaries. The Miño River is the largest river in the Galician region (Northwestern Spain), with a length of 340 km and a wide drainage basin of 17,026 km². This river has a high flow rate and is fed by many small rivers that traverse both livestock farming areas and urban areas [36]. The study area encompassed the upper basin, which includes the metropolitan area of Lugo, with approximately 98,000 residents [37], and areas dedicated to agriculture and farm production.

A total of 14 sampling points were selected for this study, focusing on the section of the Miño River between the villages of Rábade and Chantada in the province of Lugo. Eight sampling points were located at different points along the Miño River and six along its tributary, the Asma River and other streams and brooks. The study area is shown in Figure 1.

The sampling strategy was based on the locations of dairy farms. Because slurry generated by these livestock farms is generally used as fertilizer for field crops and grazing pastures in the same area, river sites near these farms were selected to investigate their impact in the Galician environment. Other sites along the Miño River were chosen to evaluate the influence of the human population, which consumes large amounts of pharmaceuticals. Thus, samples were collected at sites downstream from the discharge points of two WWTPs.

The surface water samples were collected over three seasons (late autumn, winter and spring), from November until May. This period covers the portion of the year when common crops are grown in Galicia, such as corn, potatoes, horticultural products, vineyards and forage crops. In this region, these crops are normally fertilized with slurry and manure. Antimicrobial compounds are more frequently used on livestock farms during the same period. Between November and May, animal infections become more frequent due to inclement weather conditions; consequently, prophylactic antimicrobial use is common.

### 3.2. Water Samples

The method was developed and validated using river water samples collected from the Miño River, located in Northwestern Spain. To monitor the presence of drug residues in this Galician water, 157 surface-water samples were collected in 1-L polyethylene vessels, each sample divided in duplicates of 500 mL, over a seven-month period. After collection, the samples were filtered and stored at 4 °C until extraction, which took place within 48 h after sample collection.

### 3.3. Sample Preparation and Analysis

All water samples were taken to the lab within six hours after sampling and filtered under vacuum using a 0.47-µm glass-microfiber filter (Filter-Lab, La Rioja, Spain) to remove suspended solids. The filtered samples were acidified with 0.1 N HCl to a pH of 3. Solid-phase extraction (SPE) was carried out using Strata^®^-X cartridges previously conditioned with 4 mL of methanol and 4 mL of Milli-Q water. The cartridges were eluted with 8 mL of methanol, which were collected in conical glass Pyrex^®^ tubes and later evaporated to dryness in a nitrogen stream at 45 °C. The extracts were reconstituted with 200 µL of 0.1% formic acid in methanol and stored at −18 °C until further analysis by HPLC-MS/MS. The extracts were analyzed within one week following extraction.

The samples employed for the determination of physical and chemical parameters were stored at 4 °C in the laboratory until analysis, which was carried out within one day of sampling. The physical and chemical parameters were determined following the manufacturers’ instructions for the respective kits and equipment. 

### 3.4. Chemicals, Reagents and Stock Solutions

Sulfamethazine, sulfamethoxypyridazine and trimethoprim (>98% purity) and the internal standard (IS) sulfadoxine-*d_3_* were purchased from Sigma-Aldrich (St. Louis, MO, USA). The therapeutic and chemical properties of the selected drugs are presented in Table 1. Methanol and acetonitrile (HPLC-grade, ≥99.9%) were obtained from Scharlau Chemie (Barcelona, Spain), and formic acid (>99%) was purchased from Acros Organics (Geel, Belgium). Hydrochloric acid solution (0.1 N) was purchased from Merck (Darmstadt, Germany). Purified water was prepared in-house using a Milli-Q water system from Millipore (Bedford, MA, USA) and nitrogen gas (>99.98% purity) was generated by an in-house nitrogen generator from Peak Scientific Instruments Ltd. (Chicago, IL, USA).

Each compound was accurately weighed (±0.0001 g) on an analytical balance (Ohaus^®^ GA200, Nänikon, Switzerland) to prepare individual stock solutions at a concentration of 0.6 mg·mL^−1^ in methanol. These stock solutions were mixed with 0.1% formic acid in methanol to obtain stock solutions of 1 µg·mL^−1^, which were further diluted with 0.1% formic acid in methanol to obtain standard mixtures at 12.5, 25, 50, 75, 100 and 150 ng·mL^−1^. The stock solution of the IS (sulfadoxine-*d_3_*) was prepared at 0.6 mg·mL^−1^ and was diluted with 0.1% formic acid in methanol to obtain a working solution of 1 µg·mL^−1^. All of the standard solutions were stored in the dark at −18 °C for a maximum of six months.

### 3.5. Equipment

Samples were analyzed on an HPLC-MS/MS system consisting of an HPLC model 1100 from Agilent Technologies (Waldbronn, Germany) equipped with a quaternary pump, a degasser and an auto-sampler and coupled to a mass spectrometer (MS) model API 4000™ from Applied Biosystems/ MDS Sciex (Toronto, Canada) with an integrated TurboIonSpray^®^ for molecule ionization. The software Analyst 1.4.1, also from Applied Biosystems/MDS Sciex (Toronto, Canada), was employed to acquire the data and to control the system. 

The chromatographic analyses were performed by injecting 10 µL of extract into a Synergi 2.5-µm Polar-RP 100A column (50 × 2.0 mm) connected to a Polar-RP security-guard cartridge (4.0 × 2.0 mm), both obtained from Phenomenex (Macclesfield, UK). An MS2 Minishaker vortex mixer from IKA^®^ (Staufen, Germany), a vacuum station manifold with Strata^®^-X solid-phase extraction (SPE) cartridges (60 mg, 3 mL), both from Phenomenex (Macclesfield, UK), and a Turbo Vap^®^ II evaporator from Zyrmark (Hopkinton, MA, USA) were employed for sample preparation and extraction.

Physical and chemical parameters (nitrites, ammonium, conductivity, turbidity and pH) were measured for each collected sample. These analyses utilized the following equipment and kits: Visocolor^®^ ECO Nitrite test (0.02–0.5 mg·L^−1^ NO_2_^−^) and Ammonium 3 (0.2–3 mg·L^−1^ NH_4_^+^), both from Macherey-Nagel GmbH & Co. KG (Düren, Germany), a conductivity meter model CON6/TDS6 (Hand-held Conductivity/TDS Meter) from Eutech Instruments Pte. Ltd/Oakton Instruments (Vernon Hills, IL, USA), a turbidimeter model TN-100 from Eutech Instruments Pte. Ltd. (Singapore) and a pH meter model MicropH 2000 from Crison (Barcelona, Spain). 

### 3.6. HPLC-MS/MS Method

Analytical determination was performed according to a previously reported method [38] based on solid-phase sample extraction, detection and quantification by HPLC-MS/MS. 

Analytes were separated using a gradient mixture of two components, A (0.1% formic acid in acetonitrile) and B (0.1% formic acid in water). The flow rate was 0.15 mL·min^−1^ throughout the run.

Mass-spectrometry measurements were performed using positive electrospray (ESI^+^) and pharmaceutical compounds were identified using two multiple reaction monitoring (MRM) transitions and their retention times (t_R_).

### 3.7. Statistical Analysis

The results were analyzed using the software Statgraphics Centurion XVI (StatPoint Technologies, Inc., Warrenton, VA, USA) to identify statistically significant trends in the antimicrobial concentrations. Sulfamethazine, sulfamethoxypyridazine and trimethoprim were detected at sufficiently high frequencies to be analyzed individually (Table 1).

The effects of the weather conditions, sampling site characteristics (rural and urban areas), sampling date and physical and chemical parameters (nitrites, ammonium, conductivity, turbidity and pH) were tested using one-way ANOVA (*p* = 0.05).

## 4. Conclusions

The occurrence of antimicrobials in the environment, especially in aquatic systems, has recently become a matter of concern. This study monitored sulfamethazine, sulfamethoxypyridazine and trimethoprim residues in river water samples. The results confirmed the presence of these drugs in the Galician aquatic environment; positive samples were detected at all sampling sites. The compounds were present at the ng·L^−1^ level, with a maximum concentration of 85.4 ng·L^−1^ for trimethoprim. Of the total samples analyzed (n = 314), 37% were positive. The sampling sites located downstream from WWTPs discharge points yielded the highest numbers of positive samples, most likely due to a concentration effect. 

At the site located near the collection point of a drinking-water treatment plant, 6% of the samples collected were positive for the presence of at least one of the analyzed antimicrobials, with a maximum concentration of 56.3 ng·L^−1^ for trimethoprim. 

The relationships between the concentrations of the selected pharmaceuticals in the Galician surface water and various environmental factors were statistically tested. The results showed that the concentration of sulfamethoxypyridazine depended on the sampling date and weather conditions (temperature, humidity and solar irradiation). However, this relationship was not observed for trimethoprim. 

The most important issues of concern related to the presence of these compounds in the environment are the possibility that they may exert ecotoxicological effects on non-target organisms and that they may possibly enter into the human food supply via the water cycle. 
